# Information and Support Needs Following Biomarker‐Informed ADRD Diagnoses: Insights from New IDEAS Participants in the PARADE Study

**DOI:** 10.1002/alz70858_103415

**Published:** 2025-12-26

**Authors:** Jennifer H Lingler, Jeong Eun Kim, Marita Garrett, Hector Salazar, Yuchen Zhang, Cristina de Rosa, Melissa L Knox, Megan G Witbracht, Josh D Grill

**Affiliations:** ^1^ University of Pittsburgh Alzheimer's Disease Research Center (ADRC), Pittsburgh, PA, USA; ^2^ University of Pittsburgh, Pittsburgh, PA, USA; ^3^ School of Nursing, University of Pittsburgh, Pittsburgh, PA, USA; ^4^ University of Pittsburgh School of Nursing, Pittsburgh, PA, USA; ^5^ Institute for Memory Impairments and Neurological Disorders, University of California, Irvine, Irvine, CA, USA; ^6^ University of California, Irvine, Irvine, CA, USA; ^7^ The UC Irvine Institute for Memory Impairments and Neurological Disorders, Irvine, CA, USA

## Abstract

**Background:**

As ADRD diagnoses increasingly incorporate biomarker testing, there is a pressing need to understand the experiences, challenges, and support needs of patients, and family members of those undergoing evaluations for cognitive decline. This analysis characterizes the information and support needs of racially and ethnically diverse patients and family care partners after learning a patient's brain amyloid status as part of a diagnostic evaluation.

**Methods:**

We conducted three focus groups (FGs) with persons enrolled in the Patient And family member Reactions to biomarker‐informed ADRD DiagnosEs (PARADE RF1AG080591) Study, which launched as an add on to the New IDEAS Study. The semi‐structured FGs were conducted over a secure video conferencing platform with trained facilitators. FG interview guides consisted of 5 open‐ended lead questions with follow‐up probes aiming to explore participants' information and support needs after receiving amyloid PET results. Verbatim transcripts of FG recordings were analyzed using thematic analysis.

**Results:**

The FGs included a mix of both cognitively symptomatic patients (*n* = 11) and care partners (*n* = 9) and all were referred from New IDEAS. Participants’ mean age was 68.6 years, 80% were college educated, and 7 self‐reported being, or caring for someone, amyloid positive. Participants’ average national ADI percentile was 53.4 (range:9‐96, SD:28.5). Half identified as Black or African American, 25% as Hispanic, and 25% as non‐Hispanic White. Information needs consistently fell into the categories of: test‐related, diagnosis‐related, and treatment‐related. Discussion of the latter included spontaneous mention of amyloid‐lowering therapies in two of the focus groups with participants voicing both accurate (e.g., need to treat early) and inaccurate (e.g. requires “blood transfusions”) understandings. Support needs spanned the domains of: emotional, spiritual, family, financial, and care‐related resources. Satisfaction with information and support provided by clinicians varied widely and included evidence of the need for decisional support (e.g., “We chose not to have the infusion. I don't know if that was a good choice or not”).

**Conclusions:**

Patients and families navigating biomarker‐informed ADRD diagnoses report a broad array of information and support needs, including those well documented in studies conducted before the advent of biomarker testing as well as emerging concerns related to novel therapeutics.

